# Additions of trastuzumab to preoperative chemotherapy or chemoimmunotherapy for patients with potentially resectable stage III to IV_B_ HER2-positive gastric cancer

**DOI:** 10.3389/fimmu.2025.1624943

**Published:** 2025-07-18

**Authors:** Xuchen Zhang, Yulong Tian, Huiyun Wang, Shanai Song, Yunqing Chen, Ning Liu, Chuantao Zhang, Xiao Huang, Haitao Jiang, Helei Hou

**Affiliations:** ^1^ Department of Oncology, The Affiliated Hospital of Qingdao University, Qingdao, China; ^2^ Department of Gastrointestinal Surgery, The Affiliated Hospital of Qingdao University, Qingdao, China; ^3^ Department of Pathology, The Affiliated Hospital of Qingdao University, Qingdao, China

**Keywords:** HER2-positive gastric cancer, preoperative therapy, trastuzumab, immune checkpoint blockade, tumour regression grade

## Abstract

**Background:**

Whether the addition of trastuzumab to chemo(immuno)therapy for the preoperative treatment of patients with potentially resectable HER2-positive gastric cancer has clinical benefits remains to be explored. This real-world observational study was designed to evaluate the efficacy and safety of trastuzumab plus chemo(immuno)therapy for neoadjuvant or conversion therapy in patients with potentially resectable HER2-positive gastric cancer.

**Methods:**

We retrospectively collected the clinical data of treatment-naïve patients with potentially resectable stage III to IV_B_ HER2-positive gastric cancer who received preoperative therapy prior to D2 gastrectomy. The main outcomes of interest included tumour regression grade (TRG), treatment-related adverse events (TRAEs), and event-free survival (EFS).

**Results:**

A total of 40 patients were included in the analysis, specifically, 27 patients (67.5%, 95% CI 0.520-0.799) received preoperative trastuzumab plus chemo(immuno)therapy, and 13 patients (32.5%, 95% CI 0.201-0.480) received chemo(immuno)therapy. All these patients subsequently underwent D2 gastrectomy. Regarding surgical outcomes, TRG0/1 rates were 33.3% (95% CI 0.186-0.522) in the trastuzumab-containing treatment group and 15.4% (95% CI 0.043-0.422) in the chemotherapy/chemoimmunotherapy group. Regarding safety, 66.7% (95% CI 0.478-0.814) of patients in the trastuzumab-containing treatment group and 61.5% (95% CI 0.355-0.823) of patients in the chemotherapy/chemoimmunotherapy group experienced preoperative TRAEs. The probabilities of EFS were not statistically significant between the two groups by the last follow-up.

**Conclusion:**

Additions of trastuzumab to preoperative chemotherapy or chemoimmunotherapy resulted in a TRG0/1 rate of 33.3% among patients with potentially resectable HER2-positive gastric cancer, and the combined regimen exhibited a favourable safety profile.

## Introduction

Gastric cancer (GC) remains a severe medical burden with high morbidity and mortality worldwide (1), and human epidermal growth factor receptor-2 (HER2)-positive GC is a distinct subtype with high invasiveness. HER2-positive GC accounts for approximately 10% to 20% of all GC cases, and the prognosis of patients with HER2-positive GC is dismal (2). For patients with potentially resectable HER2-positive GC, the malignant biological behaviours of the tumour worsen surgical outcomes and prognoses; thus, exploring the optimal preoperative treatment options for patients with potentially resectable HER2-positive GC is important.

Based on the results of the milestone phase III MAGIC trial (3), perioperative chemotherapy and D2 radical gastrectomy has become the standard treatment regimen for patients with locally advanced GC. In recent years, the combination of neoadjuvant programmed cell-death receptor (ligand)-1 (PD-1/PD-L1) blockade and chemotherapy has resulted in higher pathological complete response (pCR) rates than neoadjuvant chemotherapy alone, according to the phase III KEYNOTE-585 study (4) and the MATTERHORN study (5). However, previous studies have not specifically reported outcomes in HER2-positive cohorts, and whether the addition of HER2 blockade and/or immune checkpoint blockade to chemotherapy would have clinical benefits for patients with potentially resectable HER2-positive GC remains unclear.

For patients with unresectable or metastatic HER2-positive GC, trastuzumab plus chemotherapy is the standard first-line treatment regimen, according to the phase III ToGA trial (6). Recently, with the rapid development of immune checkpoint blockade therapies for solid tumours, the combination of pembrolizumab, trastuzumab and chemotherapy has been shown to result in promising clinical outcomes as a first-line treatment for patients with unresectable or metastatic HER2-positive GC, with significantly increased objective response rates (ORRs) and prolonged progression-free survival (PFS), as demonstrated by the phase III KEYNOTE-811 trial (7). In the preoperative neoadjuvant or conversion setting for patients with partially resectable HER2-positive GC, several studies, albeit only phase II, have investigated the efficacy and safety of preoperative trastuzumab with or without PD-1 blockade plus chemotherapy in patients with potentially resectable HER2-positive GC (8–14). According to the phase II NEOHX study (8) and HER-FLOT study (9), the pCR rates of neoadjuvant trastuzumab plus chemotherapy ranged from 8% to 22%. Additionally, the combination of PD-(L)1 blockade, trastuzumab and chemotherapy resulted in pCR rates ranging from 31% to 43% (10, 11, 14). However, the efficacy and safety of this combined regimen in patients with potentially resectable HER2-positive GC warrant further exploration in larger cohorts.

In this observational study, we retrospectively evaluated the efficacy and safety profile of the additions of trastuzumab to preoperative chemo(immuno)therapy in patients with potentially resectable HER2-positive GC.

## Methods

### Study design

We retrospectively collected clinical data from treatment-naïve, clinical stage T_3-4a_N_+_M_0_ (stage III) or stage T_any_N_any_M_1_ (stage IV_B_) HER2-positive gastric cancer patients who were treated at our centre between November 2018 and August 2024. Patients with stage IV_B_ disease in this study were considered to have partially resectable GC after multidisciplinary discussions. All procedures were approved by the Ethics Committee of the Affiliated Hospital of Qingdao University (QYFYWZLL28829, Qingdao, China). All investigations were carried out according to the principles of the Declaration of Helsinki.

The major inclusion criteria for the patients were as follows: a) a diagnosis of clinical stage T_3-4a_N_+_M_0_ (stage III) or stage T_any_N_any_M_1_ (stage IV_B_) gastric cancer with HER2 positivity; b) a lack of previous antitumour treatment; c) an Eastern Cooperative Oncology Group performance status (ECOG PS) score of 0 to 1; and d) sufficient vital organ function. The exclusion criteria for patients were as follows: a) inadequate vital organ function or systemic autoimmune disease; or b) other primary malignancies in addition to gastric cancer.

### Treatment

The included patients received trastuzumab plus chemo(immuno)therapy (trastuzumab-containing treatment group), or chemo(immuno)therapy alone (chemotherapy/chemoimmunotherapy group) as preoperative treatment. Following the final dose of preoperative treatment, tumour response was assessed, and multidisciplinary discussions were conducted to determine the feasibility of surgical resection. Subsequently, all patients underwent standardized D2 gastrectomy.

Trastuzumab was administered at the dosage of 8 mg/kg for the first cycle and subsequent 6 mg/kg, iv drip, and every three weeks. The PD-1 blockade used included one of the following regimens: sintilimab (200 mg iv drip, every three weeks), camrelizumab (200 mg iv drip, every three weeks), tislelizumab (200 mg iv drip, every three weeks) and pembrolizumab (200 mg iv drip, every three weeks). The chemotherapy regimen included standardized FLOT, SOX, XELOX, TP, TS, FOLFOX, and DCF regimens. Post-operative treatment would be continued based on the surgical outcomes and the results of multi-disciplinary treatment discussions.

### Assessments

Contrast-enhanced computed tomography (CT) and endoscopic ultrasound (EUS) were used to assess the primary tumour at baseline and the response to preoperative treatment according to Response Evaluation Criteria in Solid Tumors (RECIST) version 1.1 (15). Tumour tissue biopsies were collected both at baseline and during surgery. Surgical samples from primary tumours and lymph nodes were staged based on the gastric cancer staging system in the eighth edition of the American Joint Committee on Cancer staging manual (16).

The pathological response of the primary lesion after surgery was evaluated in accordance with the tumour regression grade (TRG) criteria (17). TRG0, TRG1, TRG2, and TRG3 were defined as no residual viable tumour cells (pCR), no more than 2% residual viable tumour cells, more than 2% but no more than 50% residual viable tumour cells, and more than 50% residual viable tumour cells, respectively. The pathological images were scanned using Nano Zoomer S210 (Hamamatsu).

Next-generation sequencing (NGS), immunohistochemical (IHC) staining with an anti-HER2 antibody, and HER2 *in situ* hybridization (ISH) were used to evaluate the expression of HER2. HER2 positivity was defined as HER2 amplification shown by NGS, an IHC staining score of 3+, or an IHC staining score of 2+ in combination with HER2 amplification confirmed by ISH (18).

The expression of programmed death ligand-1 (PD-L1) was evaluated using IHC staining with the anti-PD-L1 antibody 22C3 (Dako, Glostrup, Denmark). The combined positive score (CPS) was calculated to evaluate the number of PD-L1-positive cells, including tumour cells, macrophages and lymphocytes. Briefly, a CPS<1 indicated PD-L1 negativity, and a CPS≥1 indicated PD-L1 positivity.

The mismatch repair (MMR) status was evaluated using immunohistochemical staining with primary antibodies against MSH2, MSH6, MLH1, and PMS2. As for the microsatellite instability (MSI) status, real-time quantitative polymerase chain reaction was used to detect the amplification of five microsatellite loci: BAT25, BAT26, D5S346, D17S250, and D2S123. If two or more unstable markers were observed at these five loci, MSI-H status was defined; otherwise, the microsatellite status was defined as MSI-low (MSI-L) or MSS (19).

The observed treatment-related adverse events (TRAEs) during the preoperative treatment period were evaluated according to National Cancer Institute Common Terminology Criteria for Adverse Events (CTCAE) version 5.0.

### Outcome evaluation

The outcomes of interest were pathological tumour response and radiographic tumour response (complete response, CR; partial response, PR; stable disease, SD; progressive disease, PD). Other outcomes included the event-free survival (EFS) time (defined as the time from diagnosis to any one of the following three events: inability to undergo surgery due to disease progression, local or distant disease relapse, or death due to any cause) and observed TRAEs.

### Statistical analysis

Descriptive statistics for continuous variables were presented as either medians (interquartile ranges) or means (standard deviations), depending on data distribution. Categorical variables were summarized using frequencies and percentages with 95% confidential intervals (CIs). To assess differences between two groups, Student’s *t*-test was employed for continuous variables, while the chi-square test or Fisher’s exact test was used for categorical variables, as appropriate. A two-tailed *P* value*<*0.05 was considered indicative of statistical significance. All analyses were performed using SPSS 25.0 (IBM Corp., Armonk, NY, USA) and GraphPad Prism 9.5 (GraphPad Software, Inc., San Diego, CA, USA).

## Results

### Enrolled patients and treatment

A total of 40 patients were included in the analysis, among whom 27 patients (67.5%, 95% CI 0.520-0.799) received trastuzumab plus chemotherapy/chemoimmunotherapy (trastuzumab-containing treatment group), and 13 patients (32.5%, 95% CI 0.201-0.480) were treated with chemotherapy/chemoimmunotherapy alone (chemotherapy/chemoimmunotherapy group). Most patients in the two groups had stage III diseases, and the tumours in all the patients were considered potentially resectable after multidisciplinary discussions. Following the final dose of neoadjuvant or conversion treatment, all patients underwent standardized D2 radical gastrectomy, and the patients with stage IV_B_ disease received additional local therapy for metastatic lesions. Postoperative adjuvant treatment was administered based on the surgical outcomes such as HER2 and PD-L1 expression levels, and statuses of TMB and MSI/MMR.

11 patients (11/27, 40.7%, 95% CI 0.245-0.593) in the trastuzumab-containing treatment group were treated with the combined regimen of trastuzumab, PD-1 blockade and chemotherapy; and 4 patients (4/9, 44.4%, 95% CI 0.189-0.733) in the chemotherapy/chemoimmunotherapy group received PD-1 blockade plus chemotherapy. Trastuzumab was administered at the dosage of 8 mg/kg for the first cycle and subsequent 6 mg/kg, iv drip, and every three weeks. The details of PD-1 blockade used in these patients were: eight cases of sintilimab (200 mg iv drip, every three weeks), four cases of camrelizumab (200 mg iv drip, every three weeks), two cases of pembrolizumab (200 mg iv drip, every three weeks), and one case of tislelizumab (200 mg iv drip, every three weeks). The chemotherapy regimens used included standardized FLOT, SOX, XELOX, TP, TS, FOLFOX, and DCF regimens. **Table 1** shows the baseline characteristics and details of the treatment regimens of the patients.

**Table 1:** Demographics and baseline characteristics of the included patients.

Characteristics	Trastuzumab-containing treatment group (N=27)	Chemotherapy or chemoimmunotherapy group (N=13)	*P* value
Age (years)			0.280
Mean (Standard Deviation)	60.6 (8.77)	63.8 (8.86)	
Gender, n (%)			>0.999
Male	25 (92.6)	12 (92.3)	
Female	2 (7.4)	1 (7.7)	
Preoperative treatment, n (%)			0.730
Chemotherapy-based	16 (59.3)	9 (69.2)	
Chemoimmunotherapy-based	11 (40.7)	4 (30.8)	
Primary tumour location, n (%)			0.437
Gastro-oesophageal junction	5 (18.5)	4 (30.8)	
Non-gastro-oesophageal junction	22 (81.5)	9 (69.2)	
Tumour differentiation, n (%)			0.446
Well	0	1 (7.6)	
Moderately	10 (37.1)	4 (30.8)	
Moderately-poorly	6 (22.2)	4 (30.8)	
Poorly	11 (40.8)	4 (30.8)	
Baseline T staging, n (%)			0.400
T_2-3_	4 (14.8)	4 (30.8)	
T_4a-4b_	23 (85.2)	9 (69.2)	
Baseline N staging, n (%)			0.393
N_1-2_	21 (77.8)	12 (92.3)	
N_3_	6 (22.2)	1 (7.7)	
Baseline PD-L1 expression, n (%)			0.710
CPS<1	1 (3.7)	1 (7.7)	
1≤CPS<5	6 (22.2)	3 (23.1)	
CPS≥5	5 (18.5)	4 (30.8)	
Unknown	15 (55.6)	5 (38.4)	

In addition, a total of 10 patients completed next-generation sequencing testing, and *TP53* missense alterations were the most common co-occurred mutations.

### Clinical activity and tumour response

**Figure 1:**
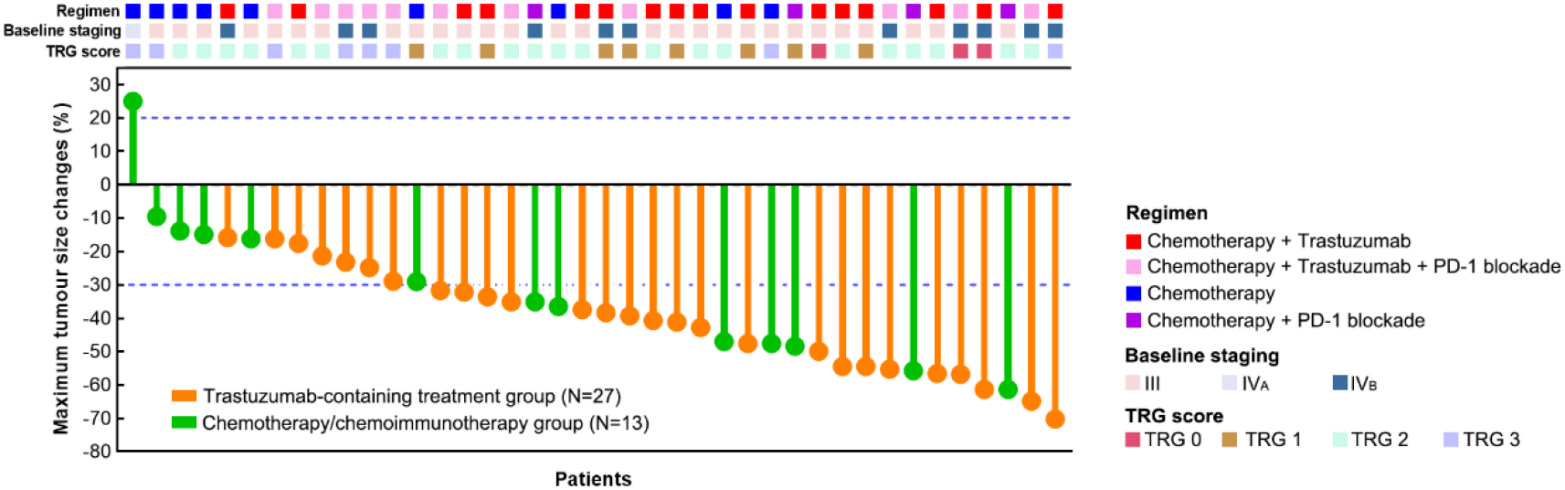
Assessments of best tumour responses to preoperative treatment based on RECIST and surgical outcomes of the patients.

According to the radiographical tumour response to preoperative treatment, all the patients in the trastuzumab-containing treatment group and most patients (12/13, 92.3%, 95% CI 0.667-0.986) in the chemotherapy/chemoimmunotherapy group had PR or SD, while one patient (1/13, 7.7%, 95% CI 0.014-0.333) in the chemotherapy/chemoimmunotherapy group exhibited PD. The ORRs were 74.1% (20/27, 95% CI 0.553-0.868) in the trastuzumab-containing treatment group, and 53.8% (7/13, 95% CI 0.292-0.768) in the chemotherapy/chemoimmunotherapy group (**Figure 1** and **Table 2**).

**Table 2:** Tumour responses and surgical outcomes.

Characteristics	Trastuzumab-containing treatment group (N=27)	Chemotherapy or chemoimmunotherapy group (N=13)	*P* value
Tumour responses (RECIST), n (%)			0.216
Partial response (PR)	20 (74.1)	7 (53.8)	
Stable disease (SD)	7 (25.9)	5 (38.5)	
Progressive disease (PD)	0	1 (7.7)	
Tumour regression grade (TRG), n (%)			0.564
TRG0	3 (11.1)	0	
TRG1	6 (22.2)	2 (15.4)	
TRG2	13 (48.2)	8 (61.5)	
TRG3	5 (18.5)	3 (23.1)	
Type of gastrectomy, n (%)			0.216
Proximal partial	2 (7.4)	2 (15.4)	
Distal partial	14 (51.9)	3 (23.1)	
Total	11 (40.7)	8 (61.5)	
Extent of resection, n (%)			-
R_0_	27 (100.0)	13 (100.0)	
Lymph nodes, mean (range)			
Harvested	23.0 (5-44)	27.9 (13-43)	0.168
Involved	1.9 (0-15)	1.5 (0-9)	0.711
Lauren’s classification, n (%)			0.627
Intestinal	5 (18.5)	4 (30.8)	
Diffused	3 (11.1)	2 (15.4)	
Mixed	10 (37.0)	5 (38.4)	
Unknown	9 (33.4)	2 (15.4)	
Post-operative PD-L1 expression, n (%)			0.858
CPS<1	1 (3.7)	0	
1≤CPS<5	6 (22.2)	3 (23.1)	
CPS≥5	10 (37.0)	6 (46.1)	
Unknown	10 (37.0)	4 (30.8)	

**Figure 2:**
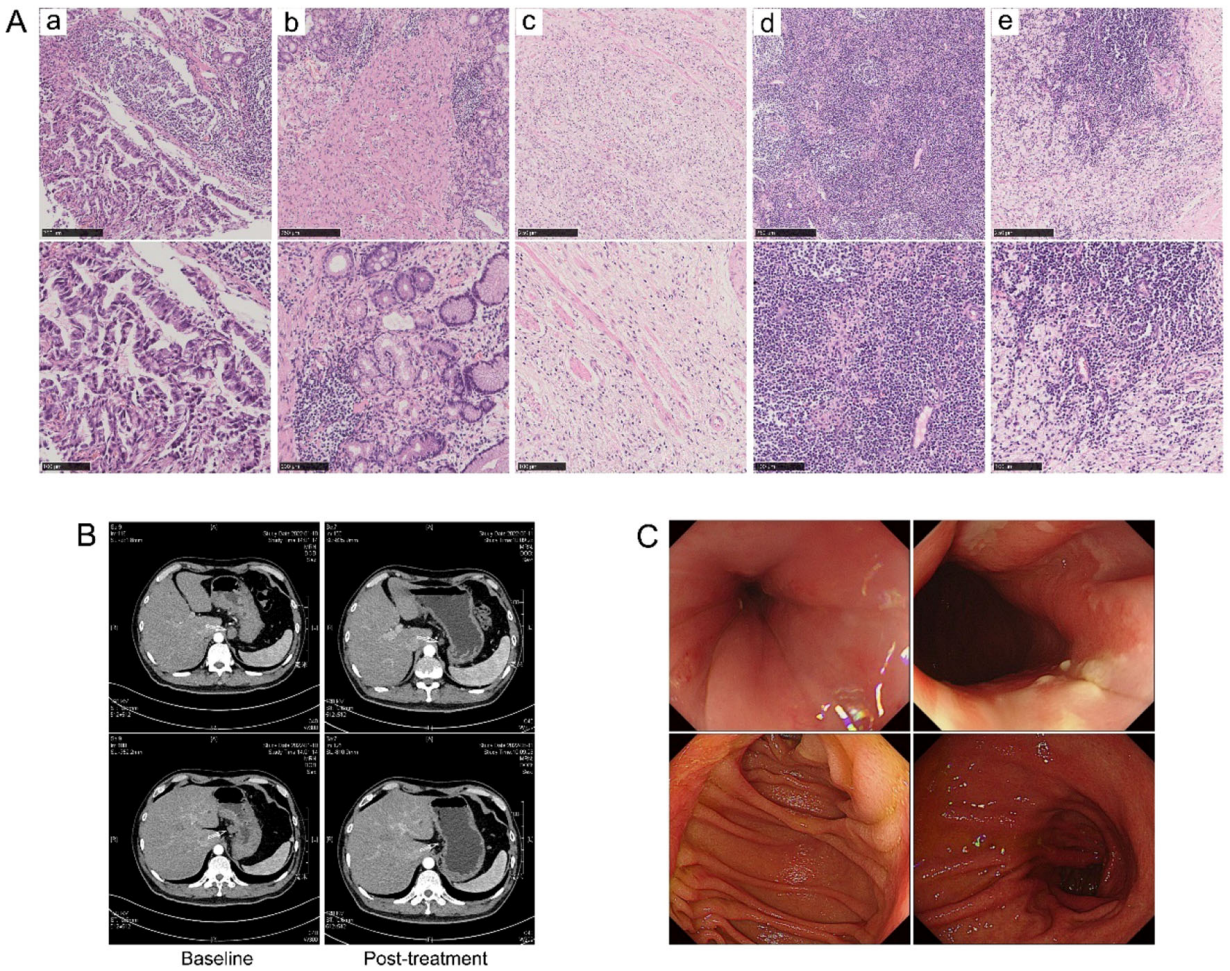
Tumour response in a patient who received preoperative treatment with trastuzumab, pembrolizumab, and XELOX chemotherapy, and subsequently achieved a pathological TRG0. **(A)** H&E staining images (a) at baseline and (b–e) post-operative. **(B)** Radiographical images at baseline and after preoperative treatment. **(C)** Gastroscopy images at 32 months post-surgery, with no evidence of recurrence observed.

R0 resection was achieved in all the patients. Three patients (11.1%, 95% CI 0.039-0.281) in the trastuzumab-containing treatment group achieved TRG0, including two baseline stage IV_B_ patients with oligometastatic lesions in the liver; while none of the patients in the chemotherapy/chemoimmunotherapy group achieved TRG0. The overall TRG0/1 rates were 33.3% (9/27, 95% CI 0.186-0.522) in the trastuzumab-containing treatment group and 15.4% (2/13, 95% CI 0.043-0.422) in the chemotherapy/chemoimmunotherapy group (*P*=0.286). **Figure 2** illustrates the tumour response in a patient who received preoperative treatment with trastuzumab, pembrolizumab, and XELOX chemotherapy, and subsequently achieved a pathological TRG0.

**Figure 3:**
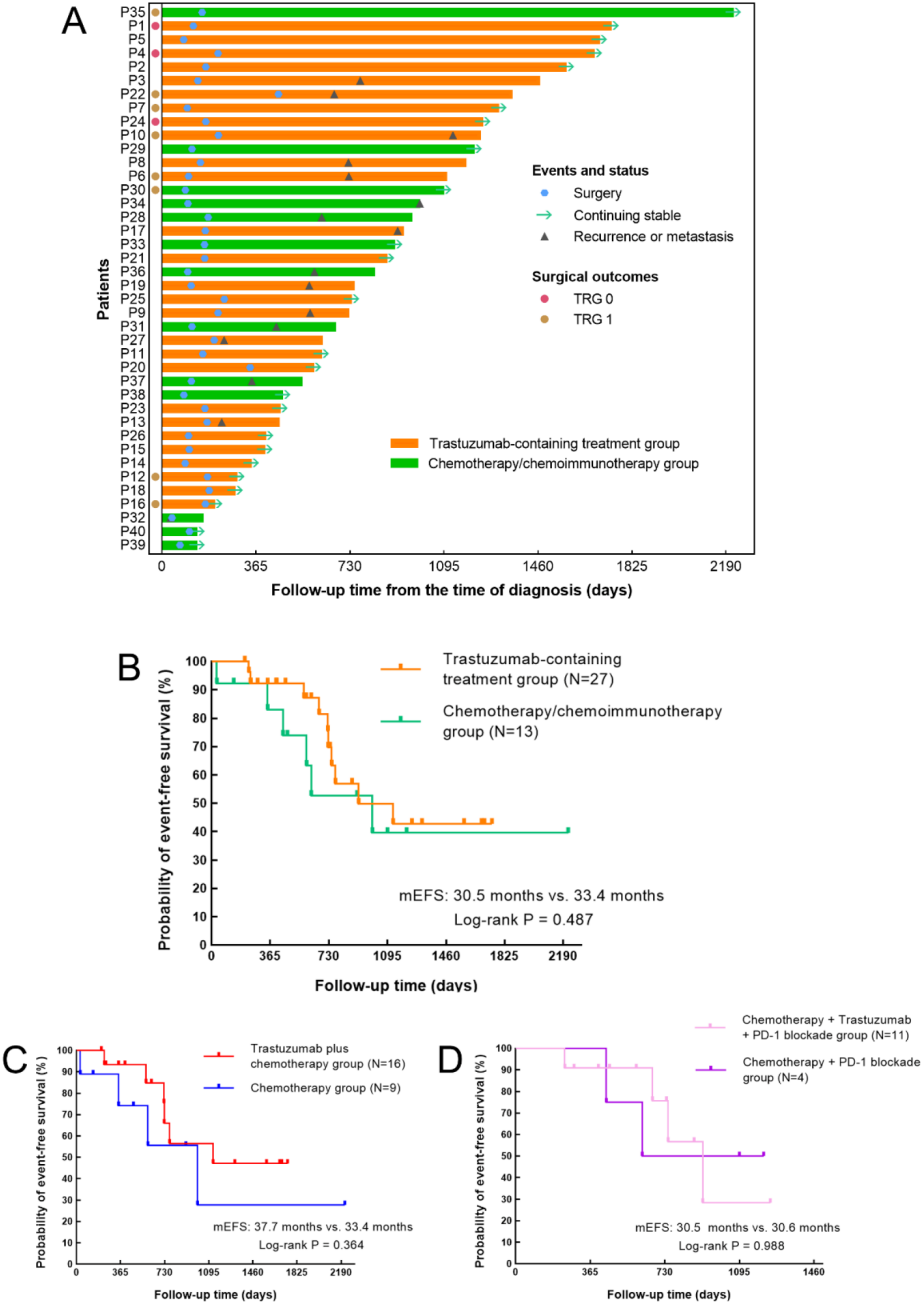
Treatment process and Kaplan–Meier curves for event-free survival (EFS). **(A)** Swimmer plot of the treatment process and follow-up of the patients from the time of diagnosis. **(B–D)** Kaplan–Meier curves for EFS **(B)** in patients receiving trastuzumab-containing treatment versus patients receiving chemo(immuno)therapy (*P*=0.487), **(C)** in patients receiving trastuzumab plus chemotherapy versus patients receiving chemotherapy alone (*P*=0.364), and **(D)** in patients receiving trastuzumab, PD-1 blockade and chemotherapy versus patients receiving PD-1 blockade plus chemotherapy (*P*=0.988).

By the last follow-up, the median follow-up time was 24.5 months in the trastuzumab-containing treatment group and 27.1 months in the chemotherapy/chemoimmunotherapy group. Ten patients (37.0%, 95% CI 0.215-0.558) in the trastuzumab-containing treatment group and six patients (46.2%, 95% CI 0.232-0.709) in the chemotherapy/chemoimmunotherapy group had reached EFS endpoints (**Figure 3A**). The median EFS time was 30.5 months in the trastuzumab-containing treatment group and 33.4 months in the chemotherapy/chemoimmunotherapy group, respectively (*P*=0.487), as shown in **Figures 3B–D**.

One patient with a surgical outcome of TRG1 underwent post-operative dynamic monitoring of minimal residual disease (MRD). The results indicated a relatively high risk of recurrence; however, after a follow-up period of 9.6 months, no evidence of recurrence was observed. A longer follow-up period is required to further assess this patient’s survival outcome.

### Safety and feasibility

Generally, preoperative treatment regimens containing trastuzumab plus chemotherapy or chemoimmunotherapy have shown a favourable safety profile. 18 patients (66.7%, 95% CI 0.478-0.814) in the trastuzumab-containing treatment group and eight patients (61.5%, 95% CI 0.355-0.823) in the chemotherapy/chemoimmunotherapy group experienced TRAEs of any grade, with anaemia and decreased neutrophil count being the most common. Other TRAEs observed in the patients were well tolerated and could be managed by symptomatic treatment. **Table 3** shows the details of the preoperative TRAEs.

**Table 3:** Preoperative treatment-related adverse events in the included patients.

TRAEs, n (%)	Trastuzumab-containing treatment group (N=27)		Chemotherapy or chemoimmunotherapy group (N=13)	
	Grade 1-2	Grade 3-4	Grade 1-2	Grade 3-4
Haematological system				
Neutrophil count decreased	6 (22.2)	7 (25.9)	3 (23.1)	1 (7.7)
Anaemia	9 (33.3)	2 (7.4)	2 (15.4)	2 (15.4)
Platelet count decreased	1 (3.7)	1 (3.7)	0	1 (7.7)
Digestive system				
Anorexia	1 (3.7)	0	1 (7.7)	0
Vomiting	3 (11.1)	0	1 (7.7)	0
Diarrhoea	1 (3.7)	0	0	0
Increased aminotransferase level	2 (7.4)	2 (7.4)	1 (7.7)	0
Skin				
Rash	1 (3.7)	0	0	0
Immune-related AEs				
Hepatitis	0	1 (3.7)	0	0
Myositis	0	1 (3.7)	0	0

## Discussion

The optimal treatment options for patients with potentially resectable HER2-positive GC have been under explorations. In patients with locally advanced HER2-positive breast cancer, the efficacy and safety of preoperative trastuzumab-based therapy have been confirmed in several randomized controlled trials (20–22). However, for patients with potentially resectable HER2-positive GC, whether the addition of trastuzumab with/without PD-1 blockade to preoperative chemotherapy has clinical benefits is not clear. In our current study, we observed that preoperative trastuzumab plus chemo(immuno)therapy as neoadjuvant or conversion therapy resulted in a TRG0/1 rate of 33.3% in patients with potentially resectable stage III-IV_B_, HER2-positive GC, and this combination approach was well tolerated. Encouragingly, this combined regimen also showed promising outcomes in patients with baseline IV_B_ disease, with two patients achieving TRG0. Although the limited sample sizes made it difficult to show statistically significances, these results still indicated that preoperative trastuzumab-containing therapy can potentially benefit patients with potentially resectable HER2-positive GC.

According to the phase II NEOHX study (8) and HER-FLOT study (9), trastuzumab can be safely added to neoadjuvant chemotherapy, and this combination approach resulted in pCR rates ranging from 8.3% to 22.2% in patients with locally advanced HER2-positive GC. In the randomized phase II JCOG1301C study, grade 1a/1b surgical efficacy was achieved in 50% of patients in the trastuzumab plus S-1/CDDP cohort (12) (**Table 4**). In our study, among the patients treated with preoperative trastuzumab plus chemotherapy, 12.5% (2/16) achieved TRG0 and 31.3% (5/16) achieved TRG1, with an overall TRG0/1 rate of 43.8% (7/16), consistent with the results in the above trials. In addition, the efficacy and safety of perioperative trastuzumab, PD-(L)1 blockade, and chemotherapy in patients with potentially resectable HER2-positive GC are being investigated in several ongoing phase II trials (10, 11, 23), and the pCR rates range from 31.3% to 42.9% according to the preliminary results, as shown in **Table 4**. In our study, the TRG0 rate was 9.1% (1/11) in patients receiving preoperative trastuzumab, PD-1 blockade, and chemotherapy, and the overall TRG0/1 rate of this cohort was 18.2% (2/11), which was inferior to that in the abovementioned studies, possibly owing to the relatively late stage and the limited sample size.

**Table 4:** Summary of clinical trials focusing on perioperative treatment of trastuzumab plus chemotherapy with/without PD-1 blockade in patients with partially resectable, HER2-positive gastric cancer.

Clinical trial	Phase of trial	Regimen	Number of patients	R0 resection rates	pCR rates^†^	Incidences of grades 3–4 TRAEs
NEOHX study (8, 33)	Phase II	XELOX+ trastuzumab	36	77.8%	8.3%	33.3%
HER-FLOT study (9)	Phase II	FLOT+ trastuzumab	58	93.3%	22.2%	27.6%
JCOG1301C study (12)	Randomized, Phase II	Arm A: S-1/CDDP; Arm B: S-1/CDDP with trastuzumab	A: 22; B: 24	A: 91%; B: 92%	A: 23%; B: 50%**^†^** (*P*=0.072)	Unknown
NCT03950271 (2022 ASCO) (10)	Single-arm, Phase II	CAPOX+ SHR1210+ trastuzumab	22	100%	31.3%	22.7%
NCT04819971 (2023 ESMO) (11)	Single-arm, Phase II	DOS+ trastuzumab+ tislelizumab	12	100%	42.9%	8.3%
NCT04661150 (14)	Randomized, Phase II	Arm A: CAPOX+ trastuzumab with atezolizumab; Arm B: CAPOX+ trastuzumab	A: 21; B: 21	Unknown	A: 38.1%; B: 14.3% (*P*=0.079)	A: 57.1%; B: 66.7%
AIO STO 0321 study (13)**^‡^**	Single-arm, Phase II	FLOT+ pembrolizumab+ trastuzumab	30 (estimated)	/	/	/
NCT05218148**^‡^**	Single-arm, Phase II	SOX+ trastuzumab+ sintilimab	44 (estimated)	/	/	/
NCT05715931**^‡^**	Single-arm, Phase II	FLOT+ trastuzumab+ toripalimab	30 (estimated)	/	/	/

CAPOX/XELOX, capecitabine and oxaliplatin; FLOT, 5-FU, leucovorin, oxaliplatin and docetaxel; DOS, Docetaxel, S-1 and oxaliplatin; SOX, S-1 and oxaliplatin; CDDP, cisplatin.

**^†^** In JCOG1301C trial, the results of pathological response were displayed as grade 1a/1b in accordance with Japanese classification.

**^‡^** These trials are still ongoing and have not disclosed related results.

Given the heterogeneity in PD-L1 expression and MSI/MMR status among GC patients, it is crucial to consider these biomarkers when selecting optimal treatment strategies. In HER2-positive GC, the phase III KEYNOTE-811 trial demonstrated that patients in the CPS≥1 subgroup had significantly higher benefits of overall survival after treatment with pembrolizumab, trastuzumab and chemotherapy, while those in the CPS<1 subgroup hardly gained clinical benefits (24), highlighting the differences in tumour immune microenvironment features between PD-L1 positive and negative patients. However, evidence regarding perioperative treatment for HER2-positive GC patients remains limited. Additionally, for GC patients with MSI-high or MMR deficiency, neoadjuvant treatment with ipilimumab plus nivolumab has been recommended (25); while the percentage of MSI-high or MMR deficiency status in HER2-positive GC is relatively low. In our study, among the 11 patients who received preoperative trastuzumab, PD-1 blockade and chemotherapy, both of the patients who achieved TRG0/1 had a CPS of 1. None of the patients were MSI-H or MMR deficiency status. The predictive role of the PD-L1 CPS and MSI/MMR in patients with potentially resectable HER2-positive GC still warrants further investigation.

In the neoadjuvant setting for patients with locally advanced HER2-positive breast cancer, postneoadjuvant treatment HER2 status conversion might predict the risk of relapse (26). However, the role of the loss of HER2 expression after preoperative treatment in HER2-positive GC patients remains unclear. In our study, four patients in the trastuzumab-containing treatment group exhibited loss of HER2 positivity after preoperative treatment. Their TRG scores were one case of TRG1 and TRG3, respectively, and two cases of TRG2; while none of them had experienced relapse by the last follow-up. Further studies are required to explore the roles and mechanisms of HER2 conversion after preoperative treatment in patients with HER2-positive GC.

HER2 amplification occurs in 23-38% of patients with hepatoid adenocarcinoma of the stomach (HAS) (27, 28). Mechanistic evidence indicates that PD-1 blockade-based immunotherapy might also be effective in patients with HAS (29). However, the optimal treatment options for patients with locally advanced HAS with concurrent HER2 amplification remain under debate. In our study, the patient with HAS developed immune-related hepatitis and myositis after one cycle of neoadjuvant PD-1 blockade plus chemotherapy, and consequently, he had to discontinue PD-1 blockade treatment. The TRG score of the patient was TRG2 after subsequent neoadjuvant treatment with trastuzumab and chemotherapy. This further indicates the importance of exploring the underlying mechanisms and optimizing treatment regimens for patients with locally advanced HAS and concurrent HER2 positivity.

RECIST has been widely applied in the evaluation of preoperative treatment efficacy in several solid tumours (15), but it seems inadequate for the evaluation of patients with potentially resectable GC. The dissimilar assessments of RECIST and TRG (17) might lead to inconsistencies in radiological and pathological evaluations. In the current study, the results of radiographical tumour responses evaluated by RECIST and pathological tumour responses evaluated by TRG in some patients were inconsistent. With the emergence of Positron Emission Tomography (PET) Response Criteria in Solid Tumours (PERCIST) (30) and its applications in several recent studies of neoadjuvant immunotherapy (31, 32), the detection of tiny lesions and quantification of metabolic activity are gradually maturing. In addition, the detection of serum biomarkers may provide insights. Robust methods for evaluating the efficacy of these preoperative treatments in patients with potentially resectable GC are needed.

There are several limitations of this study. First, the nonrandomized design might lead to inevitable bias, and the relatively limited sample size could result in a lack of power. In addition, the follow-up time was not sufficient to obtain exhaustive survival outcomes. Finally, various agents were used for PD-1 blockade and for chemotherapy; thus, conclusions on the effectiveness of a certain regimen could not be drawn. More researches are needed to explore the optimal preoperative treatment strategies for patients with potentially resectable HER2-positive GC.

## Conclusion

This study implied that trastuzumab could be safely added to preoperative chemo(immuno)therapy, and this regimen induced a TRG0/1 rate of 33.3% in patients with potentially resectable stage III to IV_B_ HER2-positive GC. Our findings should be further validated by the ongoing clinical trials.

## Data Availability

The raw data supporting the conclusions of this article will be made available by the authors, without undue reservation.
